# Surface monitoring of *L. monocytogenes* by real-time fluorescence and colorimetric LAMP

**DOI:** 10.1007/s00253-024-13318-9

**Published:** 2024-11-12

**Authors:** Maruxa Abalo, Alexandre Lamas, Carla Teixeira, Marta Prado, Alejandro Garrido-Maestu

**Affiliations:** 1 Department of Functional Biology, University of Santiago de Compostela, Santiago de Compostela, Spain; 2 Food Quality & Safety Research Group, International Iberian Nanotechnology Laboratory, Av. Mestre José Veiga S/N, 4715-330 Braga, Portugal; 3 Food Hygiene, Inspection and Control Laboratory (Lhica), Department of Analytical Chemistry, Nutrition and Bromatology, Veterinary School, Campus Terra, University of Santiago de Compostela, 27002 Lugo, Spain; 4 Laboratory of Microbiology and Technology of Marine Products (MicroTEC), Instituto de Investigaciones Marinas (IIM), CSIC, Eduardo Cabello, 6, 36208 Vigo, Spain

**Keywords:** *Listeria monocytogenes*, Surface analysis, Loop-mediated isothermal amplification, Naked-eye detection, Stainless steel

## Abstract

**Abstract:**

*Listeria monocytogenes* is a major foodborne pathogen affecting developing, and developed countries. The analysis of food contact surfaces in food industries is key for better controlling this pathogen. The current study focused on the development, optimization, and evaluation of a rapid and simple method for the detection of *L*. *monocytogenes* on stainless steel surfaces, suitable for decentralized setups, taking advantage of Loop-mediated isothermal amplification (LAMP). This was accomplished using a general pre-enrichment broth (TSB), with a simple DNA extraction based on a chelating resin, and final isothermal amplification. Two different detection strategies were tested, real-time fluorescence and naked-eye colorimetric, which were evaluated after 5, 7, and 24 h of pre-enrichment. Regardless the detection chemistry selected, after 5–7 h of pre-enrichment, 10^3^–10^4^ CFU/cm^2^ were needed to obtain a positive result, while after 24 h, it was possible to detect concentrations below 10 CFU/cm^2^. Within each given time, all the performance parameters calculated, relative sensitivity, specificity, and accuracy, reached values higher than 80–90%; likewise, a Cohen’s k of concordance with a culture-based approach higher than 0.8. Overall, the most sensitive assay can be performed in roughly 25 h. This time-to-result outperforms commercial kits with the added value of specifically detecting *L*. *monocytogenes* instead of *Listeria* spp.

**Key points:**

•* Real-time fluorescence and naked-eye colorimetric, were compared for the novel assay.*

•* An LOD50 of 3.4 CFU/cm*^*2*^* and 4.2 CFU/cm*^*2*^* was calculated for the two assays.*

•* Three pre-enrichment times were compared providing 24 h better results.*

**Supplementary Information:**

The online version contains supplementary material available at 10.1007/s00253-024-13318-9.

## Introduction

*Listeria monocytogenes*, the causative agent of human listeriosis, is one of the most important foodborne pathogens due to its high mortality rate; in 2021, it was reported to be of 13.7% in the European Union (EFSA and ECDC 2022), but it can reach values as high as 20–30% (Allerberger and Wagner 2010; Abril et al. 2021). This bacterium is highly resistant to harsh environmental conditions, it is able survive, and even grow over a wide range of pH values (4.3–9.8) and temperatures (0.5–45 °C), and it can stand high NaCl concentrations (20% w/v) as well as low water activities (a_w_ 0.91). All these properties make *L*. *monocytogenes* particularly problematic in Ready-To-Eat (RTE) food products (Warriner and Namvar 2009).

Over the last years, the incidence of this pathogen has been quite stable, with around 2000–2500 cases confirmed per year in Europe, even though in 2021 there was a 14% increase of reported cases compared to 2020 (EFSA and ECDC 2022). The official method for the detection of this pathogen relies on the ISO standard 11,290 (ISO 2017), and a plethora of alternative methods have been reported in the last few years aiming to overcome the existing limitations this standard presents, from turnaround time to subjectivity of results interpretation. To this end, the most popular approaches rely on molecular methods, more specifically, those based on nucleic acid amplification like PCR and qPCR (Rodríguez-Lázaro et al. 2005; Rossmanith et al. 2006), but also others taking advantage of isothermal DNA amplification (Liu et al. 2015; Busch et al. 2022), or even more recently based on DNA sequencing (Yang et al. 2020; Azinheiro et al. 2021, 2022b; Commichaux et al. 2021). In addition to the development of specific assays, due to the relevance, and challenges, this microorganism presents, some effort has also been made in the development of specific sample processing to improve its recovery, concentration, and purification for downstream applications which may include immunomagnetic separation, matrix lysis, among other procedures (Rossmanith et al. 2007; Duodu et al. 2009; Gattuso et al. 2014; Rodríguez-Lázaro et al. 2014; Mao et al. 2016). However, studies focusing on the recovery and detection of this pathogen from food contact surfaces are scarce, even those analyzing the its presence in environment (Muhterem-Uyar et al. 2015; Capo et al. 2020), even though it is known that cross-contamination through contact surfaces is the most common route of transfer of the bacteria to foodstuffs (Wilks et al. 2006; Fagerlund et al. 2021). Finally, it is worth to note that some of the assays currently available for the identification of this pathogen on surfaces, either are complex to perform/interpret, in many cases are still fully culture-based delaying time to results, or lack specificity and so, additional confirmation steps are needed upon having a positive result (Gómez et al. 2012; Capo et al. 2020; Lane et al. 2020). Considering the current scenario in regards to surface analysis of *L*. *monocytogenes*, the aim of the current study was to develop an economic and simple to perform assay for the specific monitoring of *L*. *monocytogenes* on surfaces. To accomplish this goal, a novel Loop-mediated isothermal amplification (LAMP) assay was developed and two different detection strategies, real-time fluorescence and naked-eye colorimetric, were compared against a classical, culture-based approach.

## Materials and methods

### Media and bacterial strains

*L*. *monocytogenes* WDCM 00021, purchased from the Spanish Type Culture Collection (CECT) and recommended as reference strain in the ISOs 11,290 and 11,133 (ISO 2003, 2017), was selected as type strain for all the spiking experiments. To this end, fresh cultures were prepared by resuspending a single colony in 4 mL of Nutrient Broth (NB, Biokar diagnostics SA, France) and incubating this suspension overnight at 37 °C. The fresh cultures were one 100-fold serially diluted in NB, and plated on Tryptic Soy Yeast Extract Agar (TSYEA, Biokar diagnostics SA, France) and the plates were incubated overnight at 37 °C. These plate counts were used to determine the concentration of viable bacteria spiked in every single sample. In addition to this, *Escherichia coli* WDCM 00013, also purchased from the CECT, was selected for mixed spiking experiments. Fresh *E*. *coli* cultures, and determination of reference values of viable bacteria, was performed as for *L*. *monocytogenes* but instead of plating on TSYEA, the dilutions were plated on Tryptic Soy Agar (TSA, Biokar diagnostics SA, France). Other than these two, the rest of bacteria included in the present study is summarized in Table 1.

**Table 1:** Strain list

Species	Reference	LAMP result	Species	Reference	LAMP result
*L. monocytogenes*	CM3	+	*Salmonella enterica* sv. Typhimurium	WDCM 00031	−
	CR1	+	*Salmonella enterica* sv. Enteritidis	UB	−
	Chicken	+	*E. coli*	C179 − 12*	−
	WDCM 00021	+		T4/97*	−
	Lhica CO55.1	+		LMV_E_2*	−
	Lhica CO55.2	+		LMV_E_3*	−
	Lhica ch	+		LMV_E_7*	−
	CECT 4032	+		LMV_E_4*	−
*L. innocua*	CECT 4050	−		EF129*	−
	CUP 1325	−		AMC 76	−
	CECT 5376	−		CECT 5941	−
	ATCC 33090	−		WDCM 00014	−
*L. ivanovii*	ATCC 12119	−		WDCM 00013	
*L. seeligeri*	ATCC 35967	−	*C. lari*	AMC 250	−
*C. difficile*	CECT 531	−	*C. coli*	UM	−

*WDCM* World Data Centre for Microorganisms reference, *ATCC* American Type Culture Collection, *CUP* Catholic University of Porto, *UM* University of Minho, *UB* University of Bristol, *CECT* Spanish Type Culture Collection. *Strains supplied by the National Institute for Agricultural and Veterinary Research (INIAV). AMC strains belong to the Collection from the Institute of Applied Microbiology—ASMECRUZ. Lhica: Food Hygiene, Inspection and Control Laboratory

The analysis of the stainless steel coupons included a pre-enrichment in Tryptic Soy Broth (TSB, Biokar diagnostics SA, France), and result confirmation was performed by streaking a loopful of the pre-enriched samples on COMPASS Listeria (COMPASS, Biokar diagnostics SA, France). The plates were incubated at 37 °C for 24–48 h. Note that this medium has the same composition as ALOA, selective media recommended in ISO 11290 for the isolation of *L*. *monocytogenes* from food and feed samples.

### DNA extraction

Two different procedures were followed for the extraction of bacterial DNA depending if pure bacterial cultures or pre-enriched surface samples were analyzed.

### Pure cultures

A fresh culture of every microorganism detailed in Table 1 was prepared as described above; 1 mL was taken and centrifuged at 16,000 × g for 2 min. Carefully, the supernatant was discarded and the pellet was resuspended in 200 μL of TE 1 × (10 mM Tris–HCl, 1 mM EDTA, pH 7.5). The bacterial suspension was thermally lysed at 99 °C for 10 min at 1400 rpm in a Thermomixer comfort (Eppendorf AG, Germany). Finally, the suspension was centrifuged for 5 min at 16,000 × g at 4 °C. The supernatant was transferred to a new tube. DNA samples were stored at 4 °C or – 20 °C for short- and long-term storage respectively, until needed.

The concentration of the DNA extracts was measured in a Qubit™ 4 Fluorimeter (Invitrogen™, Carlsbad, CA, USA), and the purity was assessed attending to the 260/280 and 260/230 absorbance ratios, measured in a NanoVue™ Plus Spectrophotometer (GE Healthcare Europe GmbH).

### Surface samples

Three different DNA extraction protocols were tested to determine which one performed best for the surface analysis. These were evaluated with and without a lysozyme pre-treatment, making a total of six potential protocols. Three independent aliquots were taken for each protocol. In all the cases, 1 mL of a fresh culture of *L*. *monocytogenes*, prepared as detailed in in the first section of “Materials and methods,” was taken and centrifuged at 16,000 × g for 3 min. The supernatant was discarded and the pellet was used for the evaluation. In cases where the lysozyme treatment was performed, 20 µL of a 20 mg/mL lysozyme solution, prepared in TE 2 × with 1.2% of Triton X100, was added to the pellet and were incubated at 37 °C for 10 min in a Thermomixer comfort at 1400 rpm. Once the incubation was completed, it was directly used for subsequent DNA extraction protocols. The selection of the final protocol to be used was based on the quality and concentration of the DNA, as well as the results obtained in real-time LAMP which was performed in triplicate.

### Direct thermal lysis

To the pellet obtained as detailed above, or the lysozyme suspension if this pre-treatment was done, 200 µL of TE 1X were added and directly incubated at 99 °C and constant agitation at 1400 rpm for 10 min. Once the lysis was completed, the samples were centrifuged at 16,000 × g and 4 °C for 3 min.

### Chelex™ 100

The pellet was resuspended in 200 µL of a 6% (w/v) Chelex™ 100 suspension (Bio-Rad Laboratories, Inc., USA) by vortexing. The samples were placed in a Thermomixer comfort set at 56 °C for 15 min under constant agitation (1400 rpm) and this was followed by a thermal lysis of the bacteria at 99 °C for 10 min and constant agitation at 1400 rpm. Lastly, the samples were centrifuged for 3 min at 16,000 × g and 4 °C.

### PrepSEQ™

When using the PrepSEQ™ Rapid Spin Sample Preparation Kit (Applied Biosystems, Foster City, CA, USA), the initial centrifugation was directly performed in the column supplied within the kit (if the lysozyme treatment was applied, the enzyme was added to this pellet). To the pellet, 5 µL of lysis buffer and 5 µL Proteinase K were added; the mixture was incubated at 56 °C for 30 min with agitation at 1400 rpm in a Thermomixer comfort; this was followed by 10 min at 99 °C and 1400 rpm, and finally 250 µL of DNase/RNase free water were added and the samples were centrifuged for 3 min at 16,000 × g and 4 °C.

### LAMP primer design

A new set of primers was designed targeting the *hly* gene of the reference genome NC_003210 of *L*. *monocytogenes* with PrimerExplorer V5 (https://primerexplorer.jp/e/index.html). For comparison purposes, the inner primers FIP and BIP were purchased with and without polyT linker separating F2 from F1c and B2 from B1c respectively, see Table 2.

**Table 2:** LAMP primers

	Sequence (5′ → 3′)	Final concentration (nM)
F3	TTC AAA AGC TTA TAC AGA TGG AA	200
B3	AAG CTA AAC CAG TGC ATT C	200
FIP	TGA ACA ATT TCG TTA CCT TCA GGA T *tttt* TCG ATC ACT CTG GAG GAT AC	1000
BIP	GGA GCG AAA ACA ATA AAA GCA AGC T *tttt* GCG TAA ACA TTA ATA TTT CTC GC	1000
LF	CAT CCC AAG AAA TGT TGA ATT GAG C	800
LB	TCG TCC ATC TAT TTG CCA GGT A	800
hly-P3F*	CGC AAC AAA CTG AAG CAA AGG A	200
hly-P3R*	CGA TTG GCG TCT TAG GAC TTG C	200
hly-P3P*	^FAM^ – CAT GGC ACC/ZEN/ACC AGC ATC TCC G – ^IABkFQ^	150

Primers designed using the *hly* gene of NC_003210, RefSeq retrieved from NCBI. In FIP and BIP the “*tttt*” represents the polyT linker introduced and tested, see Supporting Information Fig. S1. *Primers and probe from Roumani et al. (2021)

### LAMP reaction setup

Two different LAMP assays were included in the current study; on the one hand, a real-time fluorometric one, for now on real-time LAMP, and on the other hand a colorimetric one, from now on colorimetric LAMP. The differences among these assays relied on the usage of a different master mix, and the fact that the real-time LAMP also included ROX as a passive reference dye, while the colorimetric LAMP did not implement such dye.

The final reaction volume was 25 µL composed of 15 µL of GspSSD2.0 Isothermal Mastermix (ISO-004), or Visual detection RT Isothermal Mastermix (ISO-010RT-VIS) both purchased from OptiGene (OptiGene Ltd., Horsham, UK) for the colorimetric assay. The final primer concentration was 1000 nM, 200 nM, and 800 nM for FIP/BIP with polyT linker, F3/B3, and LF/LB respectively; the reaction was supplemented with 1% pullulan (TCI Chemicals Co., Ltd.) and 6 µL were added as DNA template. The remaining volume was filled with DNase/ RNase free water (Thermo Fisher Scientific, Inc., Waltham, MA, US).

The reactions for the real-time LAMP assay were run at 66 °C for 30 min with fluorescence acquisition every 30 s (60 cycles). Once the amplification step was completed, melt curve analysis was performed consisting in heating at 95 °C for 1 s, 80 °C for 20 s, and reheating up to 95 °C with increments of 0.015 °C and continuous fluorescence acquisition. This assay was performed in a QuantStudio™ 5 System and analyzed with QuantStudio™ Design & Analysis Software v1.5.1 (Applied Biosystems™, Foster City, CA, USA) that automatically calculated the cycle of quantification (Cq). The amplification temperature of the colorimetric LAMP was also performed at 66 °C but for 60 min instead of 30 min for better color discrimination, and was performed in a Veriti™ Thermal Cycler (Applied Biosystems™, Foster City, CA, USA).

In the case of the colorimetric LAMP, in addition to naked-eye assessment of the results (colorless negative vs. blue/ turquoise positive), pictures were taken with an iPhone 13Pro, and were RGB analyzed with the freely available App Pixel Picker 1.3.0.23. Five measurements were done per tube, and those with an average R value below 200 were considered positive.

### qPCR

In parallel to the novel LAMP assays, all samples were also analyzed by qPCR. To this end, the assay developed by Roumani et al. (2021) and later validated in an interlaboratory ring trial (Azinheiro et al. 2023) was selected, sequences provided in Table 2. Briefly, the final reaction volume was 20 µL composed of 10 µL of TaqMan® Fast Advanced Master Mix (Applied Biosystems™, Foster City, CA, USA), 200 nM of forward and reverse primers, 150 nM of the hydrolysis probe, 3 µL of DNA template, and the remaining volume was filled with nuclease-free water (Thermo Fisher™ Scientific, Inc., Waltham, MA, USA).

All the samples were analyzed in duplicate in a QuantStudio™ 5 System, and the thermal profile consisted on a UDG treatment at 50 °C for 2 min followed by a hot start step at 95 °C for 2 min and then 40 cycles of dissociation at 95 °C/1 s, and annealing-extension at 63 °C for 20 s.

### LAMP assay evaluation

Both, real-time and colorimetric, LAMP assays were evaluated attending to the dynamic range covered using ten-fold serial dilutions, analyzed in triplicate, of pure *L*. *monocytogenes* DNA, as well as the inclusivity and exclusivity of the assays using pure DNA of the bacterial strains detailed in Table 1 as well as in silico through nucleotide BLAST (https://blast.ncbi.nlm.nih.gov/Blast.cgi?PROGRAM=blastn&PAGE_TYPE=BlastSearch&LINK_LOC=blasthome).

### Surface sample analysis

To simulate food processing surfaces, 10 × 10 cm stainless steel coupons which were disinfected with 70% ethanol (Sigma), rinsed with DI water and after drying were UV irradiated for 15 min prior to being inoculated. Once ready, the bacteria suspension was spread on the coupons with a pipette, and they were placed in a laboratory set at 37 °C until the inoculum was dry. The same procedure was followed for mixed inoculation experiments. The bacteria were recovered using 3 M™ Sponge-Sticks (3 M, Saint Paul, MN, USA). To this end, the protocol described by Azinheiro et al. was followed. Briefly, the sponges were pre-moisten in PBS with 0.01% Tween®80, excess of liquid was squeezed out, and the sponge was passed 10 times vertically and another 10 times horizontally over the coupons, and was placed in the original bag along with 5 mL of 37 °C pre-warmed TSB (Azinheiro et al. 2020). The samples were incubated at 37 °C under constant agitation (120 rpm) in an orbital incubator (VWR) and 1 mL aliquots were taken after 5, 7, and 24 h of pre-enrichment and subjected to Chelex DNA extraction in the final method. For confirmation purposes, the pre-enriched samples were streaked on COMPASS and incubated as detailed in the first section of “Materials and methods.”

### Determination of the limit of detection

The determination of the limit of detection (LOD) was performed attending to the mathematical model described by Wilrich and Wilrich and recommended in the ISO 16140 and NordVal regulations (Wilrich and Wilrich 2009; ISO 2016; NordVal 2017). The method relies on having replicates with decreasing concentrations until a level is reached where positive and negative samples are obtained. To this end, all the samples analyzed in the current study were input in the model to calculate this parameter.

### Fit-for-purpose

One the LOD was calculated, additional samples were spiked, along with negative controls to ensure there was no previous contamination of the surfaces, and were analyzed following the method described herein and analyzed with both LAMP assays. The results obtained after LAMP analysis were compared to those expected attending to whether the samples were inoculated or not. This allowed us to classify the samples as positive or negative agreement (PA/NA) if the molecular and culture method agreed, or positive and negative deviation (PD/ND) if there were discrepancies among them. The discrepant results were compared to the results obtained after platting on COMPASS, and these samples were further re-reclassified as true positive (TP) and false positive (FP) if the molecular method was confirmed or not by the culture-based approach (see NordVal 2017). This classification of the results served to calculate the relative sensitivity, specificity, and accuracy along with the Cohen’s k or kappa following the formulae specified in the ISO 16140 and NordVal regulations (ISO 2016; NordVal 2017).

### Graphical representation and statistical analysis

The graphics and statistical analysis were all performed with GraphPad Prism v.8.0.1 software (GraphPad Software, CA, USA). The ANOVA with a Tukey post hoc was done to compare DNA extraction data with significance level of 0.05 (*p* < 0.05).

## Results

### Evaluation of the DNA extraction protocols for surface analysis

All the protocols tested, whether they had the lysozyme pre-treatment or not, produced DNA templates suitable for LAMP analyses; however, it was observed that direct thermal lysis combined with lysozyme performed was significantly worse as it presented the highest average Cq value along with the highest standard deviation and two out of the nine replicates analyzed did not amplify. This observation is in line with the fact that only one out of the three extracts reached the minimum level of quantification of the Qubit™.

**Fig. 1:**
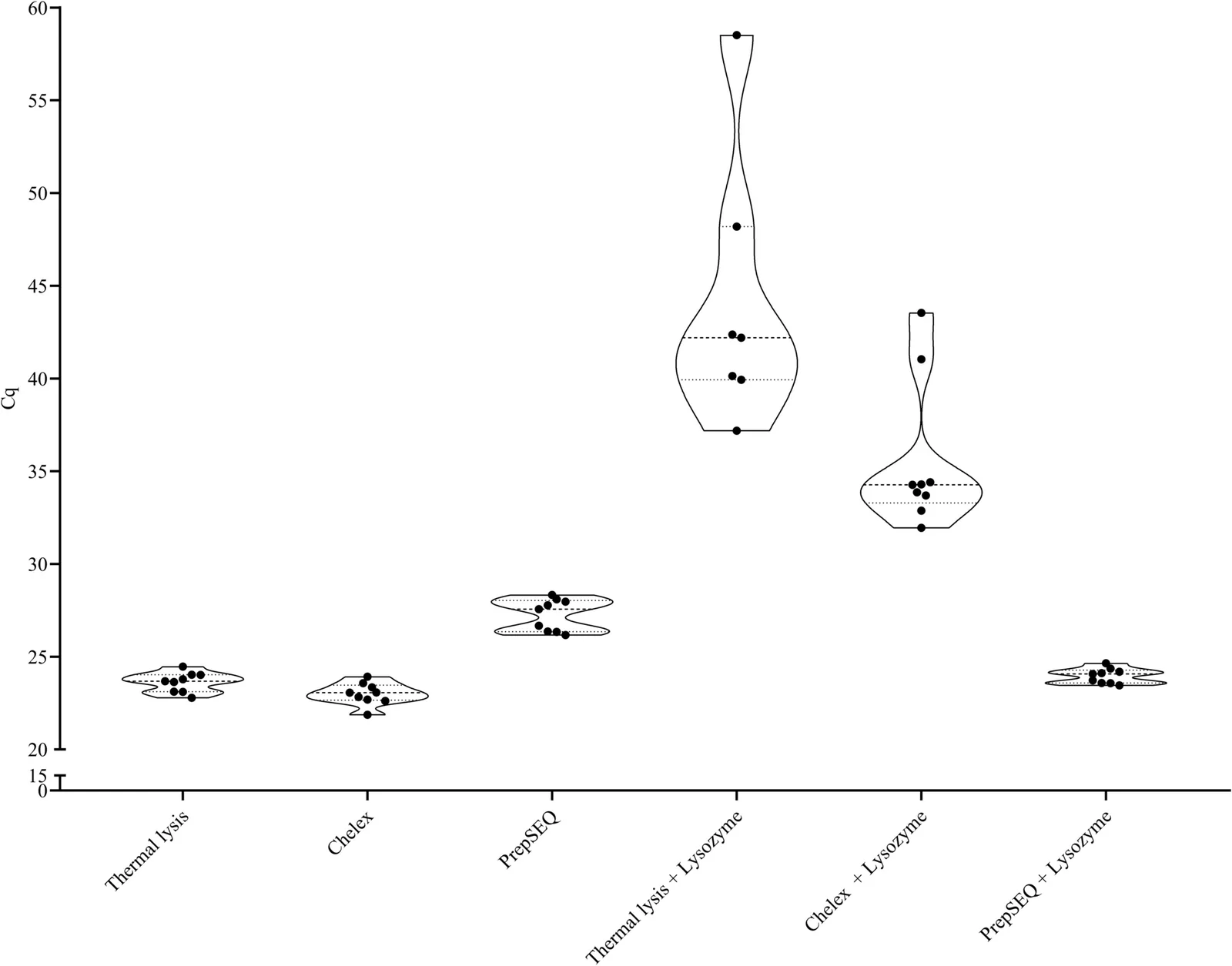
Real-time fluorescence LAMP Cq values obtained with the different DNA extraction protocols applied on inoculated surfaces. Each protocol was evaluated with and without a lysozyme pretreatment (20 mg/mL)

It was also observed that all the protocols implementing the lysozyme, except for PrepSEQ, also obtained significantly higher Cq values. Taken together, it was noticed that the lowest average Cq value was obtained with the Chelex protocol, which also obtained acceptable values of DNA purity attending to the absorbance ratios obtained, and the DNA concentration was the third highest only surpassed by the Chelex and PrepSEQ protocols implementing the lysozyme which on the other hand had worse purity values and higher Cq results. Thus, the Chelex protocol, without lysozyme pre-treatment, was selected for the final method. These data are summarized in Table 3 and Fig. 1.

**Table 3:** DNA extraction protocols comparison

Protocol	Concentration (ng/ µL)*	260/280**	260/230**	Real-time LAMP Cq
Thermal lysis	0.554 ± 0.040	2.00 ± 0.01	0.70 ± 0.01	23.62 ± 0.53
Chelex	1.403 ± 0.661	1.80 ± 0.07	0.84 ± 0.09	23.00 ± 0.60
PrepSEQ	0.327 ± 0.079	1.38 ± 0.09	0.42 ± 0.04	27.25 ± 0.86
Thermal lysis + lysozyme	0.124^+^	0.71 ± 0.01	0.16 ± 0.01	44.07 ± 7.21
Chelex + lysozyme	2.660 ± 1.455	1.59 ± 0.04	0.55 ± 0.02	35.55 ± 3.95
PrepSEQ + lysozyme	2.480 ± 0.865	0.80 ± 0.03	0.27 ± 0.09	23.97 ± 0.41

^*^DNA concentration measured with Qubit. **Absorbance ratios measured with NanoVue. ^+^Two out of the three samples were below the limit of quantification

### LAMP assay

The initial test to determine whether the addition of a polyT in the FIP/BIP primers improved the reaction indicated that this was indeed the situation, thus these primers were selected for the final assay optimization. The results may be found in Supporting Information Fig. S1.

The optimization of this novel assay was performed considering inner, outer, and loop primer concentrations, amplification temperature, supplementation with pullulan, and template volume as previously described (Roumani et al. 2021). The conditions providing the best results were 200 nM F3/B3, 1000 nM FIP/BIP with linker, 800 nM LF/LB, 1% pullulan, and 6 µL of DNA template. The best amplification temperature was determined to be 66 °C. These results are provided in Supporting Information Figs. S2 to S6.

### LAMP assay evaluation

The initial inclusivity/exclusivity test was performed in silico by analyzing with nucleotide BLAST the new set of primers designed, for which similarity with *L*. *monocytogenes* was only obtained. In addition to this, a total of 30 target and non-target strains and species were tested including 9 *L*. *monocytogenes* of different origins, 3 *L*. *innocua*, 1 *L*. *ivanovii*, and 1 *L*. *seeligeri*, acquired from official culture collections as well as wild isolates from different foods. Only positive results were obtained with the target species, and considering the melting data obtained with these target strains when analyzed with real-time LAMP, as well as those from the spiked samples, the specific Tm was experimentally determined to be 83.27 ± 0.20 °C, which was considered in subsequent experiments for real-time LAMP result confirmation.

**Fig. 2:**
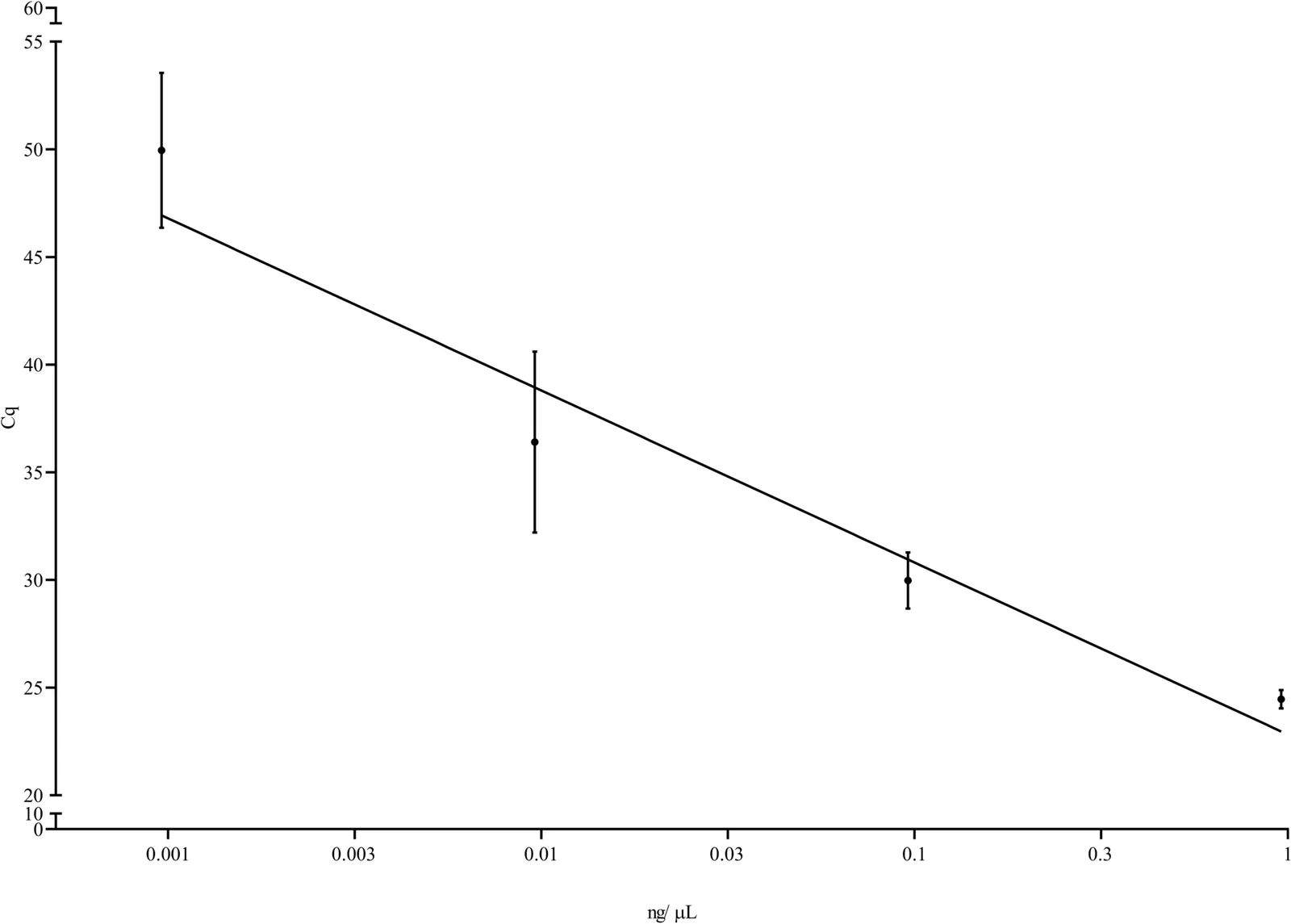

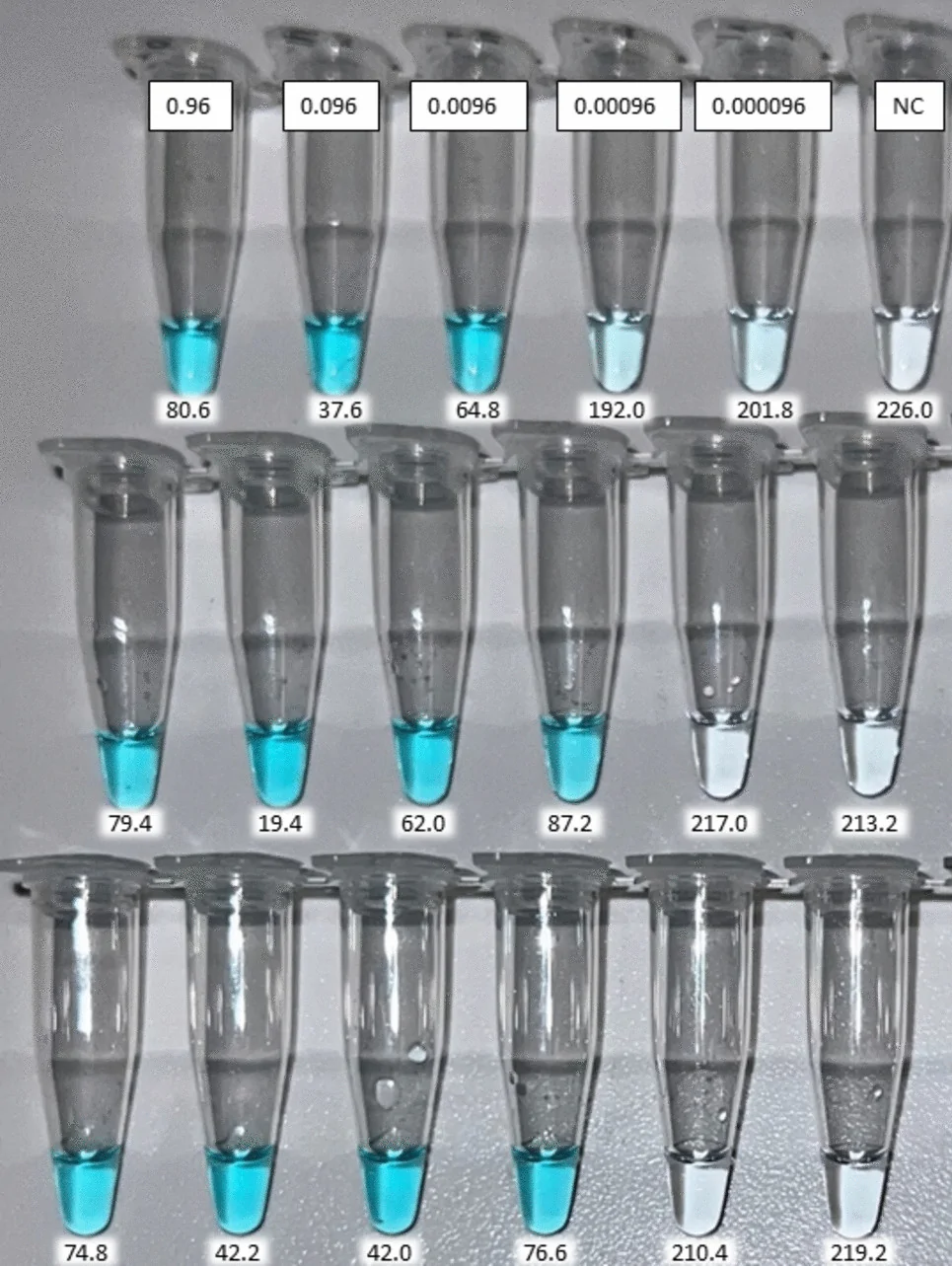
Dynamic range covered with real-time LAMP (**A**) and with colorimetric LAMP providing the average R value measured with the App Pixel Picker below each tube (**B**). In both cases, the dilutions ranged from 0.96 ng/µL down to 0.96 pg/µL

Regarding the dynamic range, a four-log serial dilution range was covered, from 0.96 ng/µL down to 0.96 pg/µL. The same concentrations were detectable with both LAMP detection strategies, fluorescence and colorimetry, as it can be observed in Fig. 2 A and B. With the lowest concentration, 0.96 pg/µL only two out of the three replicates were positive, being the same result observed with the colorimetric assay, as even though the color of this dilution was not very intense, compared to the others, by measuring the R value with the App this was classified as positive, with a value of 192.0, while the following dilution was already negative with an R value of 201.8 (R values are provided in Supporting Information Table S1).

### Determination of the LOD on spiked surface samples

The LOD was mathematically calculated inputting the data of the results obtained in the model developed by Wilrich and Wilrich. The LOD50 was determined to be 1.5 × 10^4^, 1.5 × 10^4^, and 3.4 CFU/cm^2^ after 5, 7, and 24 h of enrichment respectively when the samples were analyzed with the real-time LAMP, and 6.7 × 10^3^, 3.3 × 10^3^, and 4.2 CFU/cm^2^ with the colorimetric assay. Within each pre-enrichment time no significant differences were observed among the colorimetric and the real-time LAMP approaches. As expected, increased incubation time allowed to significantly decrease the LOD from 10^3^–10^4^ CFU/cm^2^ to less than 10 CFU/cm^2^. These results are graphically depicted in Supporting Information, Figs. S7A to S7F.

### Fit-for-purpose

Overall, 34 samples were analyzed in the current study. Considering the LOD50 calculated for each LAMP detection strategy, at 5, 7, and 24 h or pre-enrichment, 8 samples were considered for the evaluation of the real-time assay after 5 and 7 h, and 31 after 24 h. In a similar way, 9 were included in the evaluation after 5 h, 10 after 7 h, and 30 after 24 h for the colorimetric approach. As mentioned, only those samples above the calculated LOD50 were considered. In this sense, an SE and AC higher than 90% was obtained with the real-time assay at any given time, with an SP of 100% for all cases in addition to have a Cohen’s k higher than 0.90 as well. Regarding the colorimetric strategy, some additional discrepancies were observed as SE values higher than 90% were only obtained at 5 and 24 h while after 7 h, this parameter was calculated to be 83.3%. The AC of the colorimetric LAMP was again lower after 7 h compared to those of 5 h and 24 h, 90.0% compared to 100.0% and 96.7% respectively. Even though some discrepancies were identified, it is important to note that the Cohen’s k values obtained were all above 0.80. These results are summarized in Table 4. It is worth to note that no significant detrimental effect on the performance of the assays was observed in the samples co-inoculated with *E*. *coli* as, even though those samples were only detectable by LAMP after 24 h of pre-enrichment, the initial concentration of *L*. *monocytogenes* was also low (4.9 × 10^0^ and 1.1 × 10^1^ CFU/ cm^2^).

**Table 4:** Method evaluation summary for spiked samples

Pre-enrichment (h)	LOD50	N	PA	NA	PD	ND	FN	SE	SP	AC	k
Real-time LAMP											
5	1.5 × 10^4^	8	4	4	0	1	0	100.0	100.0	100.0	1.00
7	1.5 × 10^4^	8	4	4	0	4	0	100.0	100.0	100.0	1.00
24	3.4	31	24	5	0	2	2	92.3	100.0	93.5	0.91
Colorimetric LAMP											
5	6.7 × 10^3^	9	5	4	0	0	1	100.0	100.0	100.0	1.00
7	3.3 × 10^3^	10	5	4	0	1	1	83.3	100.0	90.0	0.80
24	4.2	30	23	6	0	3	1	95.8	100.0	96.7	0.95

*N*: number of samples. LOD50: Limit of Detection in CFU/ cm^2^. *PA* positive agreement, *PD* positive deviation, *NA* negative agreement, *ND* negative deviation, *FN* false negative, *SE* relative sensitivity, *SP* relative specificity, *AC* relative accuracy. k: Cohen’s kappa, interpreted as “substantial agreement” (0.61 to 0.8) and “almost complete concordance” (0.81 to 1.00) according to previous references (DG, 1991)

With the colorimetric assay, three samples spiked with 5.4 × 10^2^, 5.4 × 10^1^, and 1.1 × 10^1^ CFU/cm^2^, after 7 h of pre-enrichment, presented a faint color change and the final assessment of whether they were positive or negative was performed attending to the average R value measured with the App. In this sense, out of the three, only the sample spiked with 1.1 × 10^1^ CFU/cm^2^ was considered positive as it was the only one with an average below 200.

**Fig. 3:**
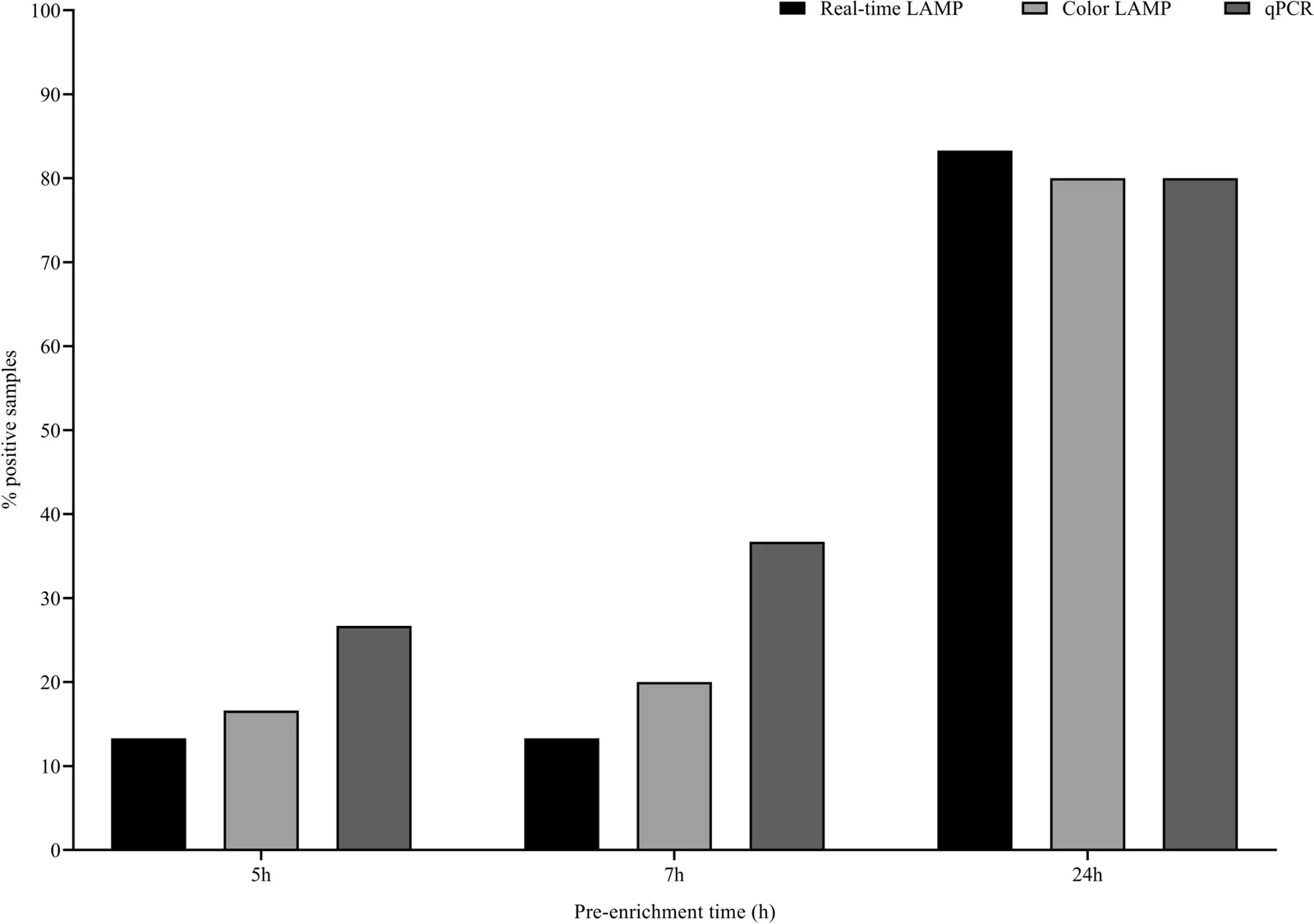
Comparison of the percentage of positive samples by real-time LAMP, color LAMP, and qPCR after 5, 7, and 24 h of pre-enrichment in TSB

When compared against qPCR, after 24 h of pre-enrichment minor differences were observed as overall there was only one sample difference among the three molecular approaches. However, the qPCR allowed to obtain a higher number of positive results after shorter periods of pre-enrichment, 5 and 7 h, where the qPCR allowed to detect, roughly, the double of positive samples compared to either LAMP assay as it can be observed in Fig. 3.

## Discussion

Food contact surface analysis is a critical step for microbial risk assessment (Miguel et al. 2012). This is of particular relevance when dealing with pathogens, such as *L*. *monocytogenes*, which can persist and multiply in harsh environments, and even resist disinfection procedures due to the formation of monospecies or mixed biofilms (Harmsen et al. 2010; Fagerlund et al. 2021). In the current study, the task to develop an easy to perform molecular test for the specific detection of *L. monocytogenes* on surfaces was addressed. In this sense, the key elements of the assay are thoroughly selected. It has been reported that sponges allow for a better recovery of bacteria from surfaces compared to swabs (Azinheiro et al. 2020), thus this sampling device was selected; however, it cannot be overseen that other factors like sampling technique, moisture, material of the sampling device, among others, may also influence bacterial recovery from surfaces (Lahou and Uyttendaele 2014; Keeratipibul et al. 2017).

Once the sampling device was selected, it was assumed that most likely a short pre-enrichment would be needed to detect low bacterial concentrations, and for this task TSB was selected as this is a classical broth present in virtually all microbiology laboratories, and which is relatively economic additionally, it was reported to allow for a faster growth of *L*. *monocytogenes* compared to other media such as brain heart infusion (Azinheiro et al. 2022a). Even though selective media may also be used for this task, their use increases the cost of the assay, and on a regular basis, they tend to extend the lag phase not making them suitable for rapid detection however, this may be the best option when heavily contaminated surfaces are being analyzed, or if there is no particular interest in a reduced time-to-result.

The following step of the final method setup was the selection of simple DNA extraction procedure. This is typically accomplished by thermal lysis. For Gram-positive bacteria, such as *L*. *monocytogenes*, enzymatic lysis are recommended when dealing with low concentrations (Kawasaki et al. 2012) but this increases the cost, turnaround time, and complexity of the assays. Upon implementation of a suitable pre-enrichment step, thermal lysis should provide satisfactory results as previously demonstrated (Rodríguez-Lázaro et al. 2004; Azinheiro et al. 2023). In the current study, three simple thermal lysis protocols were tested, direct lysis in TE 1X buffer, treatment in the chelating resin Chelex 100, and a commercial kit which implements a purification column to get rid of sample interfering material, PrepSEQ. All three were also combined tested with and without a lysozyme pre-treatment to determine whether this had or not a significant impact in the outcome of the assay. It was observed that there was not a direct correlation of the DNA concentration obtained with the named protocols and the Cq value of the real-time LAMP assay; furthermore, the implementation of the lysozyme pretreatment did not improve the results except when combined with PrepSEQ. Most likely, this observation was associated to the fact that the enzyme, and its buffer, remained in the final extract and it must not be forgotten that with the kit the DNA is diluted in a larger volume of water compared to the other two approaches, and it was reported that an excess of EDTA may have detrimental effects on the performance of LAMP assays due to the chelation of Mg^2+^ cations (Nixon et al. 2014; Moore et al. 2021). It is important to note that even though there was a delay in the amplification, positive results were still obtained thus highlighting the robustness of LAMP to the presence of typical inhibitors (Kaneko et al. 2007). Considering these results, it was decided to proceed with the protocol based on Chelex without the lysozyme pre-treatment.

The last step for the development of the method was to select an appropriate DNA amplification technique. In this sense, LAMP is an attractive option due to its isothermal nature and compatibility with many different detection strategies. In the current study, two were assessed, real-time fluorescence and end-point naked-eye detection. Due to the development of miniaturized devices, both are compatible with decentralized analyses even though the investment cost of these equipment is significantly different. Real-time detection was typically performed tracking increase in turbidity with a Loopamp device (Mori et al. 2004) but in recent years, devices capable of fluorescence monitoring like the Genie II from OptiGene have demonstrated suitable (Mckenna et al. 2017; Domesle et al. 2018, 2022) as well as the BioRanger (Diaz et al. 2019; Drais et al. 2019) among others, were developed and demonstrated to be a reliable option. If one thinks about end-point colorimetric detection, then then number of devices suitable for this purpose increases as any stable heat source is potentially suitable thus greatly expands the applicability of any given methodology (Garrido-Maestu et al. 2022). For colorimetric detection, it is crucial to select an appropriate strategy for the samples being analyzed. In an initial trial, OptiGene’s colorimetric mastermix was compared to New England Biolabs’ (NEB), being obtained better results with the first one (data not shown), and motivated the selection of the first mastermix. In a study published in 2022, Azinheiro et al. reported difficulties with NEB’s mastermix most likely due to the fact that this reagent implements phenol red, a pH-sensitive dye, to generate color change (Azinheiro et al. 2022c). Under the optimized conditions, clear color differentiation among positive and negative samples is possible; however, it was observed that for some samples, with very low DNA concentration, faint color may develop after 60 min of incubation. Typically, by extending the incubation time, faint colors become more intense and easier to interpret but this may also increase the rate of false positive results as it was reported that extended incubation times is more prone to this thus; in order to address this potential issue, a freely available cell phone App, Pixel Picker, was tested and its utility was confirmed as out of the RGB data that the App provides; the R values are always below 200 for positive samples and allowed to easily discriminate some of the samples within the present study (e.g., lowest concentration of the dynamic range). The analysis of RGB values, or their ratios, has previously demonstrated suitable for the objective assessment of color differences (Elumalai et al. 2022).

Considering that one of the initial goals of the study was to develop a rapid method, ideally allowing for same-day detection, a new LAMP assay was designed targeting the *hly* gene of *L*. *monocytogenes* which included two loop primers to speed up the reaction and potentially increasing the analytical sensitivity (Nagamine et al. 2002) as the initial assay developed in our group only had the LB primer, and was reported to obtain positive results down to 0.12 ng/µL, much higher than the value reached herein, 0.96 pg/ µL (Garrido-Maestu et al. 2018a). A later study within our group allowed to reach a similar analytical sensitivity with the mentioned assay; however, this may be associated to differences in the DNA extraction protocol applied (Roumani et al. 2021). An initial pre-screening of the inner primers confirmed that the implementation of a polyT linker improved the results as suggested by Lamas et al. (2023).

In terms of the performance of the method developed, the novel assay allowed to reach a low LOD, 3.4 and 4.2 CFU/cm^2^, for the real-time and colorimetric assays respectively, but a 24-h enrichment was needed as without it, 10^3^–10^4^ CFU/cm^2^ were needed in order to obtain a positive result highlighting the need of an enrichment step for highly sensitive detection as previously reported for other molecular methods (Broten et al. 2022; Ríos-Castillo et al. 2022). These results were in line with those reported by Azinheiro et al. after a 24-h enrichment in a selective broth, and detection by recombinase polymerase amplification (RPA) combined with lateral flow (Azinheiro et al. 2020). This isothermal technique was described by Piepenburg et al. in 2006 and has attracted the interest of the scientific community due to its performance and simple primer design; typical PCR primers may even be used (Piepenburg et al. 2006). In this regard, to address this limitation of LAMP, more primer design options are being developed in addition to Eiken’s PrimerExplorer used in the current study, NEB has launched their own platform (https://lamp.neb.com/#!/) and other specific software have been reported in the literature like LAVA and MorphoCatcher (Torres et al. 2011; Shirshikov et al. 2019; Shirshikov and Bespyatykh 2022). It is worth to note that by using LAMP, no additional consumables, such as lateral flow strips, are needed and naked-eye detection may be directly performed.

The rest of the parameters evaluated, SE, SP and AC, obtained values higher than 90%, except for the colorimetric assay after 7 h of enrichment whose values were higher than 80%, demonstrating the reliability of the novel method regardless the detection strategy followed. This was confirmed by the fact that the Cohen’s *k* values were all above 0.8 what is translated into “almost complete concordance.” In the comparison against qPCR, the results obtained were in agreement with previous studies which reported a higher analytical sensitivity of qPCR over LAMP (Zhang et al. 2011; Garrido-Maestu et al. 2018b, 2018a). Likewise, the fact that after 24 h of pre-enrichment there were no significant differences in the percentage of positive samples detected with these techniques relies on the fact that the pre-enrichment allowed to effectively increase the bacterial concentration and so, overcoming the differences in analytical sensitivity as previously commented by D’Agostino et al. and others (D’Agostino et al. 2015; Garrido-Maestu et al. 2017).

It is important to note that, even though an enrichment step is needed, this present method outperforms others currently available in the market which need 24–48 h to provide a presumptive result, which is based on color change, and lack specificity for *L*. *monocytogenes* making critical the confirmation on selective media thus further extending the duration of the method (Schirmer et al. 2012).

Attending to the results presented, the concluding remarks would be that a novel LAMP method for the rapid detection of *L*. *monocytogenes* on surfaces was developed and allowed to reliably identify the presence of this pathogen in roughly 25 h including enrichment, DNA extraction, and detection either with real-time fluorescence detection or end-point naked-eye colorimetric assessment. The best results, in terms of LOD and concordance with the reference culture-based method, were obtained after an initial pre-enrichment of 24 h highlighting the importance of this step. In addition to the turnaround time, the method demonstrated to be simple to perform and easily implementable in laboratories with low resources due to the implementation of regular, non-selective culture media, a simple thermal lysis protocol, and an easy to interpret colorimetric result assessment.

## Supplementary Information

Below is the link to the electronic supplementary material.

Supplementary file1 (PDF 752 KB)

## Data Availability

Data sets generated during the current study are available from the corresponding author on reasonable request.
