# Supplementary material for: Surface monitoring of L. monocytogenes by real-time fluorescence and colorimetric LAMP
Source: Appl Microbiol Biotechnol. 2024 Nov 12;108(1):510. doi: 10.1007/s00253-024-13318-9 (PMC11557679; doi:10.1007/s00253-024-13318-9)
Supplement: Supplementary file 1 — Supplementary file1 (PDF 752 KB) [file 253_2024_13318_MOESM1_ESM.pdf]

Supporting Information

Surface monitoring of *L. monocytogenes* by real-time fluorescence and colorimetric LAMP

Maruxa Abalo[1,2], Alexandre Lamas[3], Carla Teixeira[2,3], Marta Prado[2] and Alejandro Garrido-Maestu[2,*]

[1]Department of Functional Biology, University of Santiago de Compostela, Spain

[2]Food Quality & Safety Research Group. International Iberian Nanotechnology Laboratory, Av. Mestre José Veiga s/n, 4715-330 Braga, Portugal

[3]Food Hygiene, Inspection and Control Laboratory (Lhica), Department of Analytical Chemistry, Nutrition and Bromatology, Veterinary School, Campus Terra, Universidade da Santiago de Compostela 27002, Lugo, España

*Correspondence: alejandro.garrido@inl.int

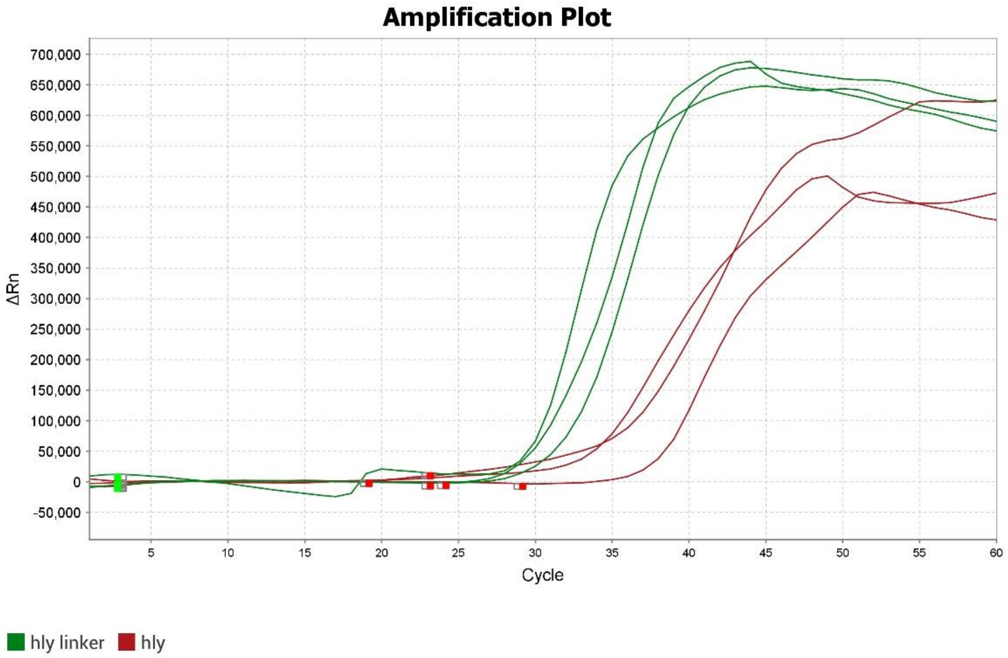

Figure S1. Comparison of FIP/ BIP with and wihout polyT linker (-T-T-T-T-). The average Cq value obtained with linker was 30.67 ± 0.97 and without linker 34.15 ± 3.45.

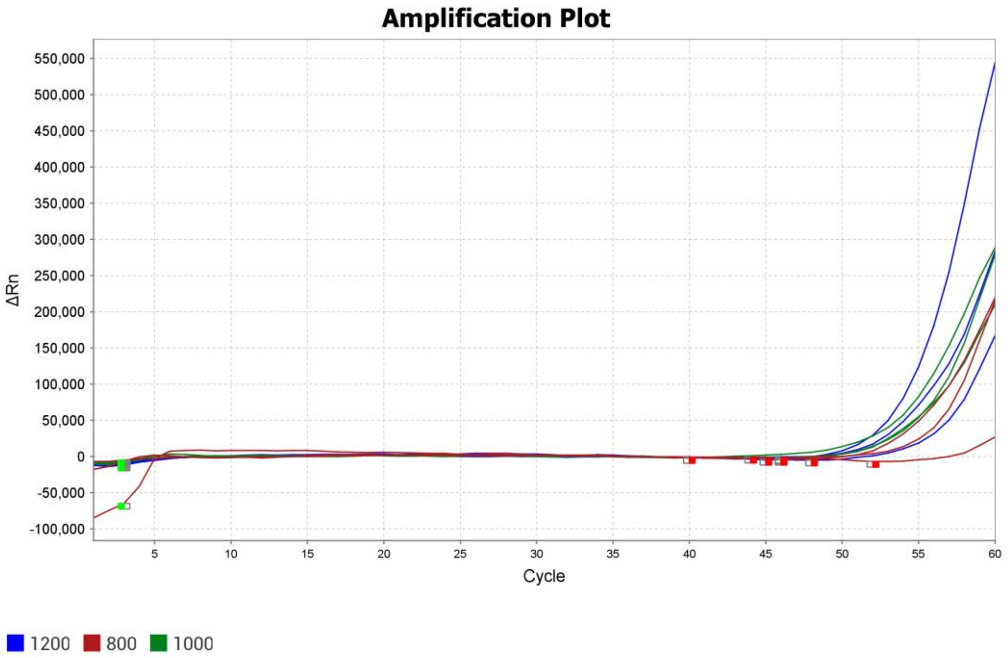

Figure S2. Comparison of 200 nM F3/B3 with 800, 1000 and 1200 nM FIP/BIP with linker. The Cq values were 55.38 ± 3.26, 53.13 ± 0.75 and 55.92 ± 2.04 for 800, 1000 and 1200 nM respectively.

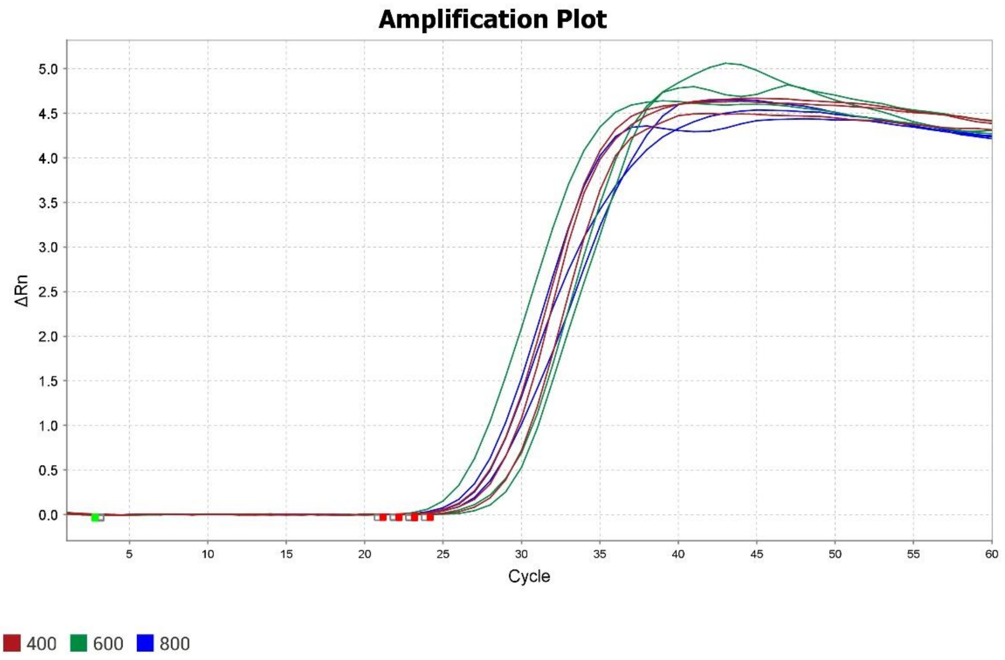

Figure S3. Comparison of LF/LB concentrations, 400, 600 and 800 nM, combined with 200 nM F3/B3 and 1000 nM FIP/BIP with linker. The Cq values were 28.73 ± 0.70, 28.23 ± 1.77 and 27.23 ± 0.42 for 400, 600 and 800 nM respectively.

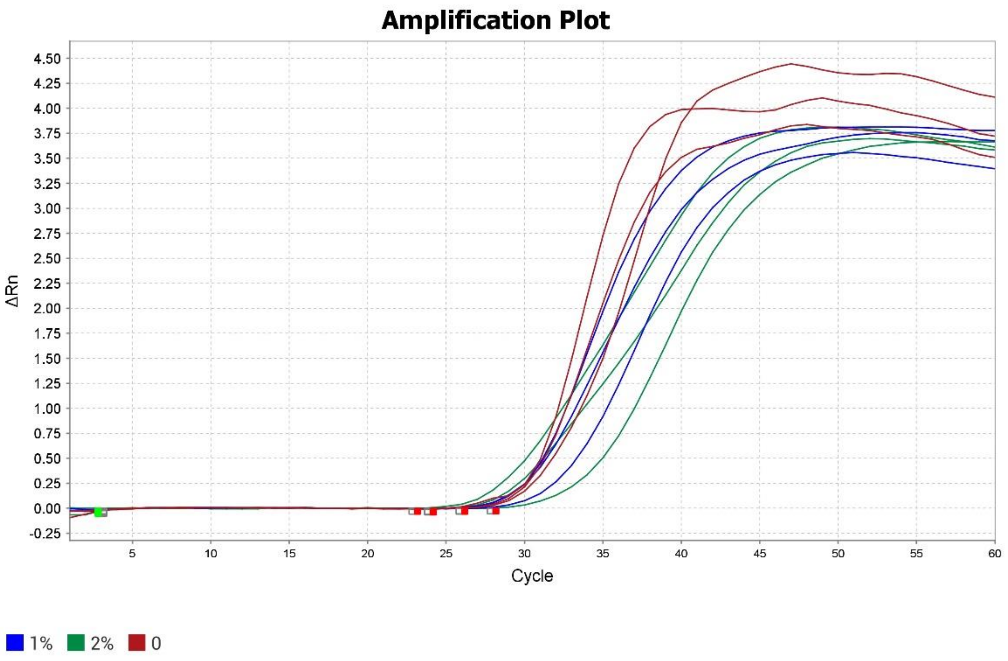

Figure S4. Evaluatoin of the effect of 1 and 2% pullulan in a reaction with 200 nM F3/B3, 1000 nM FIP/BIP with linker and 800 nM LF/LB. The Cq values were 30.84 ± 0.32, 31.12 ± 1.12 and 29.52 ± 2.41 for 0, 1 and 2% pullulan respectively.

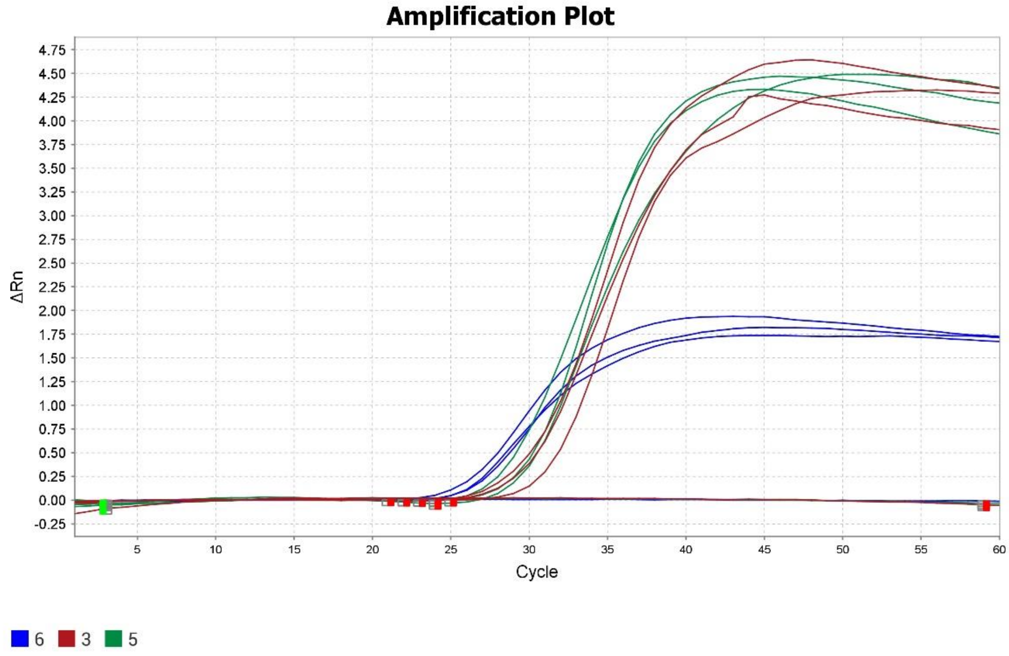

Figure S5. Template volume effect in the assay with 200 nM F3/B3, 1000 nM FIP/BIP with linker and 800 nM LF/LB and 1% pullulan. The Cq values were 30.62 ± 0.95, 29.41 ± 0.74 and 25.66 ± 0.56 for 3, 5 and 6 μL of template DNA respectively.

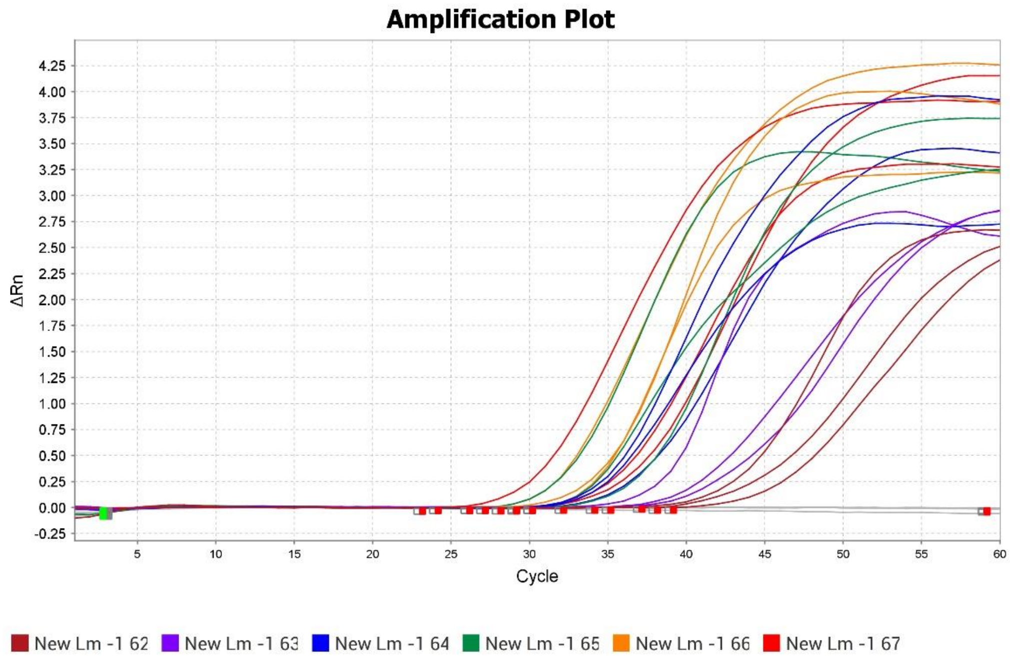

Figure S6. Evaluation of the amplification temperature effect in the assay with 200 nM F3/B3, 1000 nM FIP/BIP with linker and 800 nM LF/LB, 1% pullulan and 6 µL of template DNA. The Cq values were 43.66 ± 1.56, 39.66 ± 1.68, 34.71 ± 0.90, 33.63 ± 2.39, 32.68 ± 1.34 and 32.99 ± 3.13 at 62, 63, 64, 65, 66 and 67 °C respectively after 30 min.

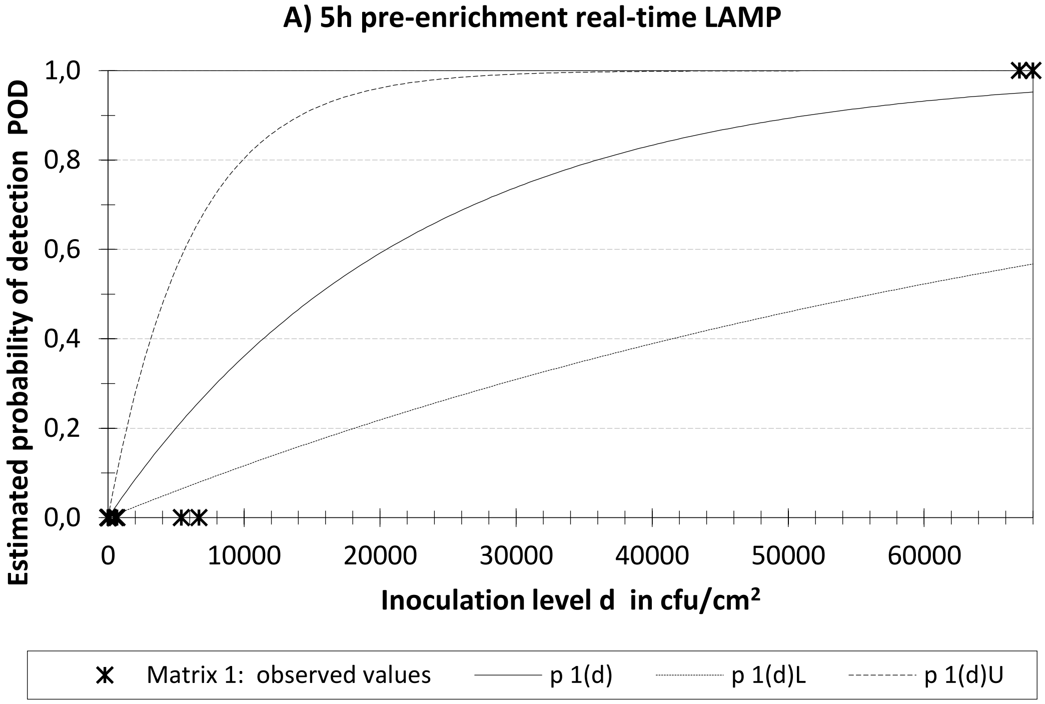

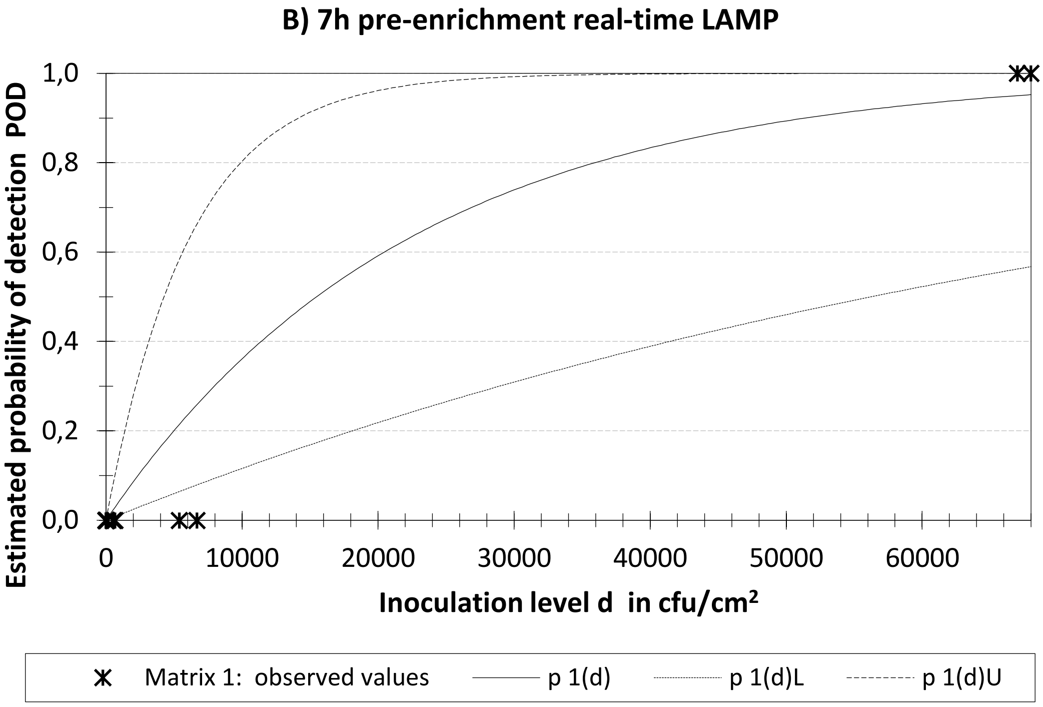
B) 7h pre-enrichment real-time LAMP
Estimated probability of detection POD
1,0
0,8
0,6
0,4
0,2
0,0
0
10000
20000
30000
40000
50000
60000
Inoculation level d in cfu/cm²
Matrix 1: observed values
p 1(d)
p 1(d)L
p 1(d)U

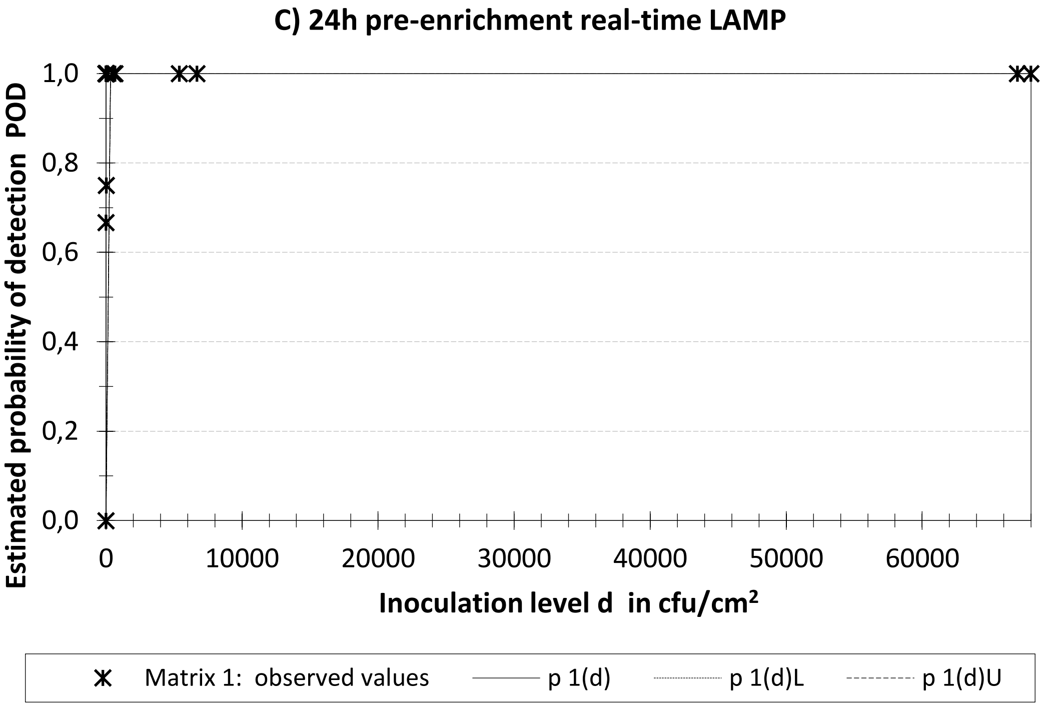
C) 24h pre-enrichment real-time LAMP
Estimated probability of detection POD
1,0
0,8
0,6
0,4
0,2
0,0
0
10000
20000
30000
40000
50000
60000
Inoculation level d in cfu/cm²
Matrix 1: observed values
p 1(d)
p 1(d)L
p 1(d)U

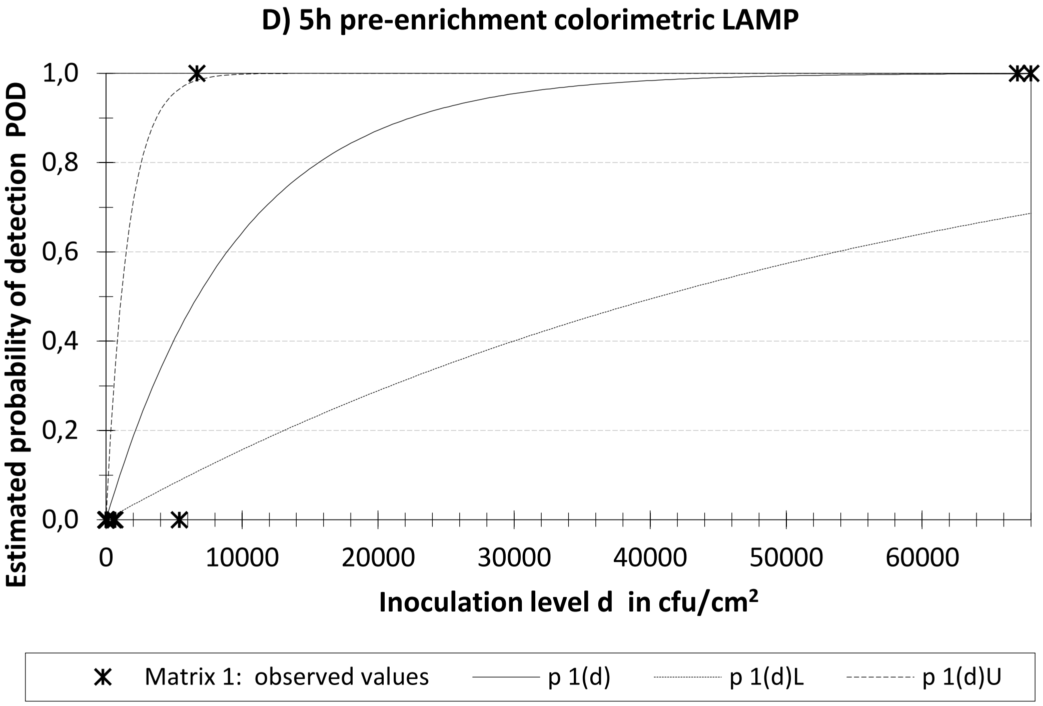
D) 5h pre-enrichment colorimetric LAMP
Estimated probability of detection POD
1,0
0,8
0,6
0,4
0,2
0,0
0
10000
20000
30000
40000
50000
60000
Inoculation level d in cfu/cm²
Matrix 1: observed values
p 1(d)
p 1(d)L
p 1(d)U

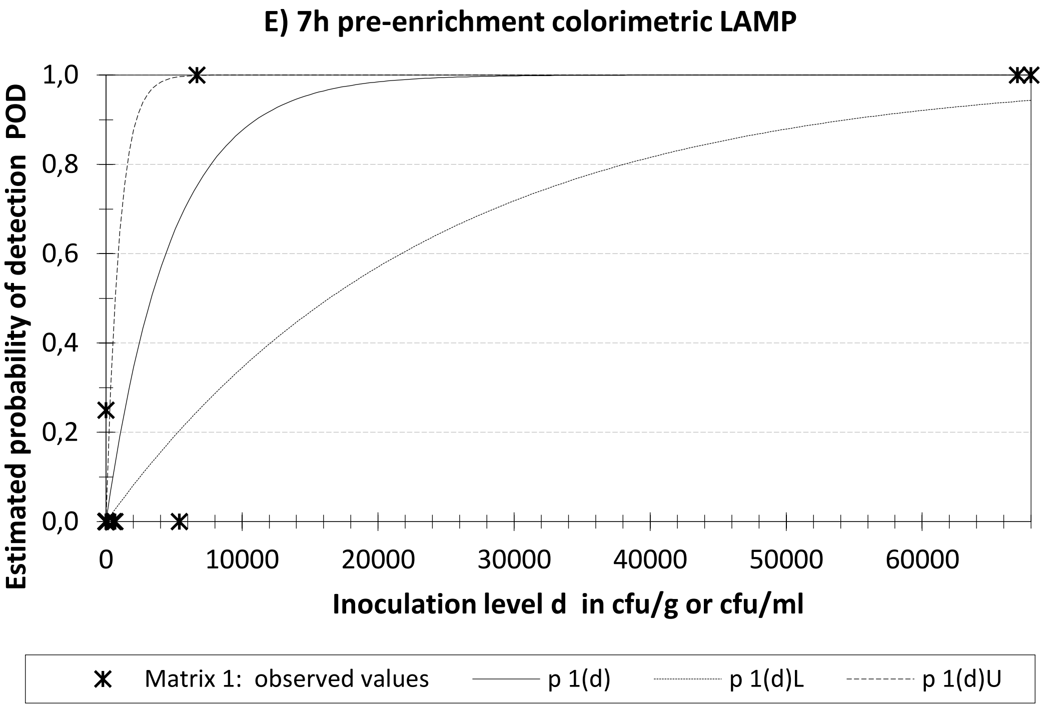
E) 7h pre-enrichment colorimetric LAMP
Estimated probability of detection POD
1,0
0,8
0,6
0,4
0,2
0,0
0
10000
20000
30000
40000
50000
60000
Inoculation level d in cfu/g or cfu/ml
Matrix 1: observed values
p 1(d)
p 1(d)L
p 1(d)U

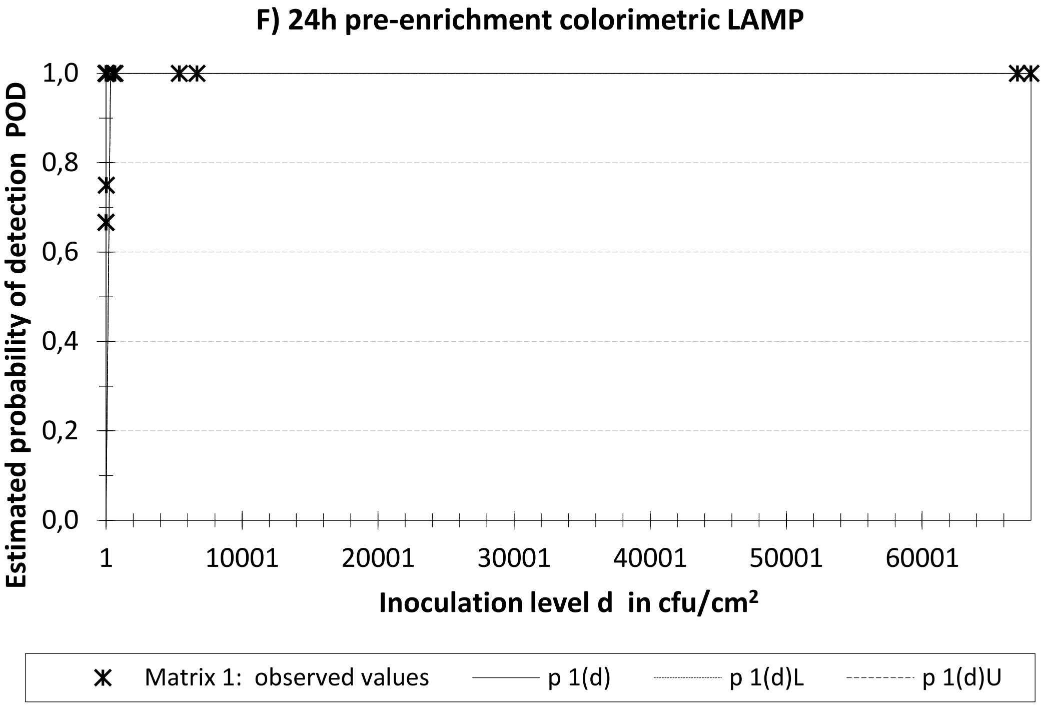

Figure S7. Representation of the LOD with the mathematical model described by Wilrich & Wilrich. A) 5 h of pre-enrichment and detection by real-time LAMP. B) 7 h of pre-enrichment and detection by real-time LAMP. C) 24 h of pre-enrichment and detection by real-time LAMP. D) 5 h of pre-enrichment and detection by colorimetric LAMP. E) 7 h of pre-enrichment and detection by colorimetric LAMP. F) 24 h of pre-enrichment and detection by colorimetric LAMP. p(d)L and p(d)U refer to the lower and upper confidence limit.

Table S1. R values from RGB measurements of the dynamic range evaluation of the colorimetric LAMP

DNA concentration (ng/ µL)	Dynamic range 1					Average	Dynamic range 2					Average	Dynamic range 3					Average
	UL	UR	BL	BR	C		UL	UR	BL	BR	C		UL	UR	BL	BR	C	
0.96	60	98	86	79	80	80.6	77	72	95	86	67	79.4	66	57	98	98	55	74.8
0.096	12	44	80	52	0	37.6	19	0	52	7	19	19.4	59	30	54	53	15	42.2
0.0096	55	49	88	75	57	64.8	47	50	92	72	49	62.0	62	29	60	48	13	42.0
0.00096	190	191	199	192	188	192.0	87	83	102	91	73	87.2	77	65	109	81	51	76.6
0.000096	208	197	210	194	200	201.8	221	215	221	222	206	217.0	216	216	210	206	204	210.4
NTC	230	228	227	218	227	226.0	217	217	221	201	210	213.2	214	197	228	234	223	219.2

UL: Up Left. UR: Up Right. BL: Bottom Left. BR: Bottom Right. C: Center. NTC: Non-Template Control

51
